# Activation of carbonic anhydrase isoforms involved in modulation of emotional memory and cognitive disorders with histamine agonists, antagonists and derivatives

**DOI:** 10.1080/14756366.2021.1891051

**Published:** 2021-03-02

**Authors:** Gustavo Provensi, Alessio Nocentini, Maria Beatrice Passani, Patrizio Blandina, Claudiu T. Supuran

**Affiliations:** a Department of NEUROFARBA, University of Florence, Section of Pharmacology and Toxicology, Firenze, Italy; b Department of Health Science, University of Florence, Section of Clinical Pharmacology and Oncology, Firenze, Italy

**Keywords:** Carbonic anhydrase, activators, cognition-related disorders, histamine receptors, agonists/antagonists

## Abstract

Carbonic anhydrases (CAs, EC 4.2.1.1) activators were shown to be involved in memory enhancement and learning in animal models of cognition. Here we investigated the CA activating effects of a large series of histamine based compounds, including histamine receptors (H_1_R – H_4_R) agonists, antagonists and other derivatives of this autacoid. CA activators may be thus useful for improving cognition as well as in diverse therapeutic areas (phobias, obsessive-compulsive disorder, generalised anxiety, post-traumatic stress disorders), for which activation of this enzyme was recently shown to be involved.

## 1. Introduction

CO_2_ is generated in most metabolic processes, being one of the simplest molecules involved in crucial physiologic processes in all life kingdoms^1^^,^^2^. The carbonic anhydrases (CAs, EC 4.2.1.1) are metalloenzymes which catalyse its interconversion to bicarbonate (Equation (1)^3–6^, generating also a proton, and thus a pH disequilibrium, which is used in most biological systems as a readily available buffering system^7^.

(1) $${\text{CO}}_{2}+H_{2}O⇌{\text{HCO}}_{3}{}^{-}+H^{+}$$

The reaction also occurs without a catalyst, but at physiological pH values it is exceedingly slow for meeting metabolic needs, as CO_2_ is a poorly water-soluble gas, which can also damage cellular components (e.g. membranes, mitochondria, etc.)^6^^,^^7^. On the other side, its conversion to water-soluble ions (bicarbonate and protons) counteracts this effect, and although interfering with the pH balance, is used to control homeostasis and metabolism, making CAs crucial enzymes in many physiological and pathological conditions^3–7^. In fact, in vertebrates at least 16 CA isoforms belonging to the α-CA genetic family are known, whereas in other organisms all over the phylogenetic tree at least seven other CA families were described so far, the β-, γ-, δ-, ζ-, η-, θ- and ι-CAs^8–14^. In humans 15 CAs are expressed, 12 of which are catalytically active: the cytosolic CA I-III, VII and XIII, the membrane-bound CA IV, the mitochondrial CA VA and VB, the secreted (in saliva and tears) CA VI, and the transmembrane CA IX, XII and XIV (the acatalytic forms are CA VIII, X and XI)^4^^,^^15–19^. many of these enzymes are drug targets, as their inhibitors show pharmacological applications for drugs treating edoema, glaucoma, obesity, epilepsy and tumours^4–6^.

The human central nervous system (CNS), as well as the choroid plexus, contains a multitude of CA isoforms, although their particular functions are not yet completely understood^17^. We will consider here mainly the CAs present in CNS, as the compounds investigated here for modulating their activity (i.e. the CA activators – CAAs) may also have interesting applications in therapy, which started to be considered only recently^20–23^. The nervous system CA isoforms comprise: the cytosolic CA I (expressed in the motor neurons in the spinal cord), CA II (present in the choroid plexus, oligodendrocytes, myelinated tracts, astrocytes and myelin sheaths); CA III (in the choroid plexus), the membrane-associated CA IV (located on the luminal surface of cerebral capillaries and associated with the blood-brain barrier, being present also in the cortex, hippocampus and thalamus). The mitochondrial CA VA is expressed in astrocytes and in neurons, whereas CA VB seems to be absent in the SNC^17^. CA VII and VIII are present in high levels throughout the cortex, hippocampus and thalamus, although CA VIII is acatalytic, whereas CA VII shows a good enzymatic activity with CO_2_/bicarbonate as substrates^4^. The acatalytic CA X is expressed in the myelin sheath, whereas CA XI (also acatalytic) is present in the neural cell body and astrocytes^17^. CA IX and CA XII are transmembrane proteins overexpressed in many neurologic cancers^18^^,^^19^, whereas CA XIII seems not to be present in the brain. CA XIV is expressed in nuclei and nerve tracts associated with pontine, medullary and hippocampal functions being also located on the plasma membrane of some neurons and on axons of mammalian brain^17^.

The most investigated CAAs are the amino acids, the biogenic amines (histamine, serotonin, catecholamines and their derivatives), and to some extent also the oligopeptides or small proteins, although these activators were less investigated^20^. The CAAs were demonstrated to participate in the catalytic cycle of the enzyme, forming enzyme-activator complexes, as described in Equation (2):^20^

(*2*) $${\text{EZn}}^{\text{2}}{}^{+}-{\text{OH}}_{\text{2}}+\text{A}⇌[{\text{EZn}}^{\text{2}}{}^{+}-{\text{OH}}_{\text{2}}-\text{A}]⇌[{\text{EZn}}^{\text{2}}{}^{+}-{\text{HO}}^{-}-{\text{AH}}^{+}]⇌\;{\text{EZn}}^{\text{2}}{}^{+}-{\text{HO}}^{-}+{\text{AH}}^{+}$$

**Figure 1.**
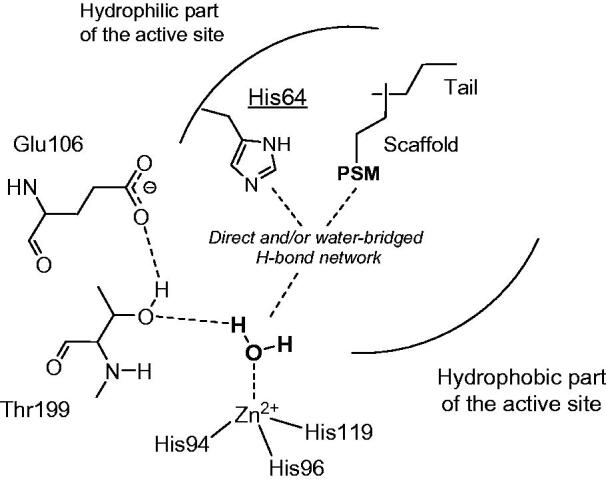
CA activation mechanisms. Activators bind in the middle of the active site and contain a proton shuttle moiety (**PSM**) of the amine, imidazole or carboxylate type with an appropriate pKa for the proton transfer processes, usually in the range of 6–8.

The activator molecule forms a complex with the enzyme, binding in an active site region distinct of that of the classical CA inhibitors^24^^,^^25^, which generally bind to the metal ion^4–6^. The activator molecule must incorporate proton shuttling moieties, which take part to the rate-determining step of the catalytic cycle, i.e. the transfer of protons from the zinc-coordinated water molecule to the external reaction medium, with formation of the nucleophilic, zinc hydroxide species of the enzyme^20^. In the wild type enzyme, this proton shuttling is achieved by residue His64 (in many CA isoforms), found within the middle of the active site cleft, and which possess the imidazole moiety able to transfer protons in the pH range of 6–8^20–24^. His64 was shown to possess two conformations: the *in* one, orientated towards the bottom of the active site, and the *out* one, orientated towards the external part of the active site, favouring thus the proton wiring^20^^,^^24^. In such processes, within the enzyme-activator complexes, the proton transfer becomes intramolecular, being more efficient compared to the intermolecular transfer to buffer molecules (which are not bound within the enzyme cavity)^20^. X-ray crystallography has been performed on several other hCA I/II – activator complexes, among which those with histamine, L- and D-His, L- and D-Phe, D-Trp, L-adrenaline as well as pyridinium derivatives of histamine^20^^,^^23–26^. A schematic representation of the activators bound to CA is shown in Figure 1.

Thus, histamine was the main compound used to obtain new CAAs^27^, but many of its rather simple derivatives as well as drugs belonging to the histamine receptors (H_1_R, H_2_R, H_3_R and H_4_R) agonists/antagonists, were not yet been investigated for their potential activating effects. Here we report the first such study, including in our investigations 28 such derivatives which have been assayed as activators of four pharmacologically significant isoforms, hCA I, II and VII (cytosolic isoforms) and hCA IV (membrane-anchored enzyme).

## 2. Materials and methods

### 2.1. Chemistry

Histamine **1** and compounds **2–30** were commercially available, highest purity reagents from Sigma-Aldrich, Milan Italy.

### 2.2. Carbonic anhydrase activation

A stopped-flow method^28^ has been used for assaying the CA catalysed CO_2_ hydration activity with Phenol red as indicator, working at the absorbance maximum of 557 nm, following the initial rates of the CA-catalysed CO_2_ hydration reaction for 10–100 s. For each activator, at least six traces of the initial 5–10% of the reaction have been used for determining the initial velocity. The uncatalyzed rates were determined in the same manner and subtracted from the total observed rates. Stock solutions of activator (0.1 mM) were prepared in distilled-deionized water and dilutions up to 0.1 nM were done thereafter with the assay buffer. The activation constant (K_A_), defined similarly with the inhibition constant (K_I_), was obtained by considering the classical Michaelis–Menten Equation (3), which has been fitted by nonlinear least squares by using PRISM 3:

(3) $$v=v_{\text{max}}/\left\{1+K_{M}/\left[S\right]\left(1+{\left[A\right]}_{f}/K_{A}\right)\right\}$$

where [A]_f_ is the free concentration of activator.

Working at substrate concentrations considerably lower than K_M_ ([S]≪K_M_), and considering that [A]_f_ can be represented in the form of the total concentration of the enzyme ([E]_t_) and activator ([A]_t_), the obtained competitive steady-state equation for determining the activation constant is given by Equation (4):

(4) $$v=v_{0}K_{A}/\left\{K_{A}+\left({\left[A\right]}_{t}–0.5\left\{\left({\left[A\right]}_{t}+{\left[E\right]}_{t}+K_{A}\right)–{\left({\left[A\right]}_{t}+{\left[E\right]}_{t}+K_{A}\right)}^{2}–4{\left[A\right]}_{t}{\left[E\right]}_{t}{}^{1/2}\right\}\right)\right\}$$

where v_0_ represents the initial velocity of the enzyme-catalysed reaction in the absence of an activator^29–32^. Enzyme concentrations in the assay system were in the range of 6.5–12.0 nM.

## 3. Results and discussion

**Figure 2.**
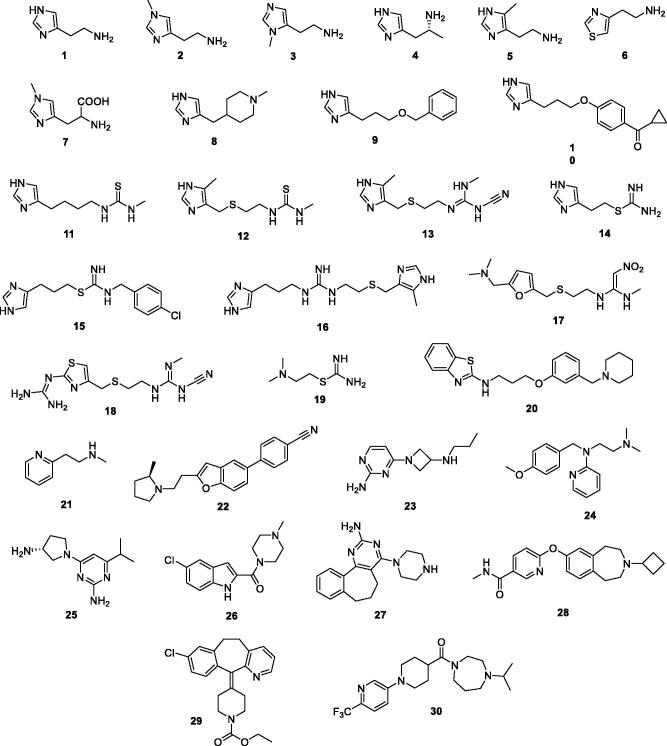
Histamine **1** and derivatives **2–30** acting as histamine receptors agonists/antagonists (for reviews see refs.^33^^,^^34^) investigated here as CAAs.

As mentioned above, histamine **1** (Figure 2) was one of the first CAAs to be investigated in detail^24^, but except for histidine (*L-* and *D-*enantiomers), other histamine derivatives were not yet assayed for their potential CA activating effects.

Considering the relatively large number of histamine receptors (H_1_R-H_4_R) as well as the huge number of agonists/antagonists developed for the management of various disorders, among which allergies, gastritis and gastric ulcers, narcolepsy, acute unilateral vestibulopathy, and atopic dermatitis^33^^,^^34^, there is a large number of compounds incorporating fragments of the histamine chemotype as well as a wealth of structural modifications which mimic this autacoid.

Some of these compounds, possessing structures **2–30** (Figure 1), were included in our study for investigating their possible CA activating effects against four pharmacologically relevant human isoforms, hCA I, II, IV and VII. The compounds were numbered according to their similarity to the lead histamine **1** and are: the H_1_R agonist 2-(2-aminoethyl)thiazole **6**; the H_2_R agonists impromidine **16** and nordimaprit **19**; the H_3_R agonists Nπ-methylhistamine **3**, α-methylhistamine **4**, methimmepip **8**, proxyfan **9**, imetit **14**, VUF16839 **23**; the H_1_R antagonists pyrilamine **24**, loratadine **29**; the H_2_R antagonists metiamide **12**, cimetidine **13**, ranitidine **17**, tiotidine **18**, zolantidine **20**; the H_3_R antagonists ciproxifan **10**, clobenpropit **15**, ABT239 **22**, GSK189254A **28**, GSK334429B **30**; the H_4_R antagonists JNJ39758979 **25**, JNJ7777120 **26**, A940894 **27**; the mixed modulators of the histaminergic system Nτ-methylhistamine **2**, 4-methylhistamine **5**, 1-methylhistidine **7**, burimamide **11**, betahistine **21**.

In some of these compounds, such as the methyl-histamines **2–5**, the thiazolyl derivative **6** or τ-methyl-His **7**, the histamine chemotype is readily observable, whereas the remaining compounds incorporate more drastic changes of the basic structure, but all of them possess moieties which can in principle shuttle protons in the pH range of 6–8 which, as mentioned earlier^20^, lead to CA activation.

**Table 1.** hCA I, II, IV and VII activation with compounds **2–30** (Figure 2) by a stopped-flow CO_2_ hydrase assay.^28^ Histamine **1** used as standard.

Compound	K_A_ (µM)^a^				Data		
	hCA I	hCA II	hCA IV	hCA VII	BBB crossing	Central action	Ref
**1**	2.1	125	25.3	37.5	–	+	^35^
**2**	0.11	8.91	3.21	2.07			
**3**	3.1	0.43	7.6	0.23			
**4**	0.12	0.082	2.91	1.25		+	^36^
**5**	0.36	5.4	5.13	0.39		+	^37^
**6**	0.87	7.45	1.02	0.7			
**7**	0.052	0.57	13.9	0.19			
**8**	3.16	5.24	4.66	0.12		+	^38^
**9**	3.15	7.66	8.01	0.52	+	+	^39^
**10**	4.29	9.9	8.12	0.11	+	+	^40^
**11**	0.88	8.39	9.07	0.43		+	^41^
**12**	0.98	8.75	9.62	1.01		+	^42^
**13**	8.79	6.3	8.54	0.59		+	^43^
**14**	4.25	8.31	8.05	1.00		+	^44^
**15**	>100	2.1	6.59	5.31	+	+	^45^
**16**	0.72	2.14	3.3	0.10			^46^
**17**	>100	>100	>100	>100		+	^47^
**18**	>100	>100	32.3	45.5		+	^48^
**19**	1.36	6.93	9.08	5.21			
**20**	>100	>100	>100	>100	+	+	^49^
**21**	>100	13.5	9.9	7.05	+	+	^50^
**22**	>100	>100	>100	>100	+	+	^51^
**23**	>100	9.82	15.9	>100		+	^52^
**24**	5.23	9.62	6.78	2.05	+	+	^53^
**25**	>100	>100	>100	>100			
**26**	>100	>100	>100	>100	+	+	^54^
**27**	>100	>100	>100	>100			
**28**	>100	>100	>100	>100	+	+	^55^
**29**	>100	>100	>100	>100	+	+	^56^
**30**	>100	>100	>100	5.06	+	+	^57^

^a^From three different assays (errors within ± 10% of the reported values). – means no BBB crossing; + means that there are evidences of BBB crossing and central action; no sign means that no literature data are available.

The following structure-activity relationship (SAR) can be worked out from the data reported in Table 1 for activation of the four isoforms hCA I, II, IV and VII:

- Compounds 17, 20, 22, 25–30 did not induce any activation of the tested CA isoforms (KAs >100 µM). Consistently, these derivatives do not possess the histamine chemotype in their structures and/or other moieties that clearly make CA activation possible. As a unique exception, compound 30 reported a 5.06 µM selective activation of hCA VII. It should be stressed that other derivatives, such as 18, 19, 21, 23 and 24, do not directly include imidazole-like scaffolds in their chemical structure, but showed however to possess significant CA activation profiles in a low micromolar range (KAs between 1.36 and 45.5 µM) and are thus included in the SAR discussion. These compounds possess however protonatable moieties of the secondary amine or guanidine type in their molecule, which like the imidazole may shuttle protons and thus act as CAAs.
- The cytosolic and ubiquitous hCA isoforms I and II were quite efficiently activated by most active derivatives (that are 2–16, 18, 19, 21, 23 and 24), in a low micromolar to submicrolar range. Intriguingly, derivatives 15, 21 and 23 did not produce any hCA I activation up to 100 µM, whereas 18 did not activates neither hCA I nor hCA II. The methylation of histamine 1 at position Nτ (2), α (4), 4 (5) and the imidazole/thiazole swap (6) increased up to one order of magnitude the hCA I activation profile (from 2.1 to 0.11 µM). The presence of an extra proton transfer group (COOH) as in 7 (Nτ-methyl-histidine) further improved 2-fold the KA (52 vs 110 nM) against hCA I compared to compound 2. The Nπ-methylation of histamine (3, KA of 3.1 µM) slightly decreased the activation efficacy of the molecule, situations also encountered for the inclusion of the aliphatic amine into a cycle (8, KA of 3.16 µM) or the amine/ether swap (as in 9 and 10, KAs of 3.15 and 4.29 µM). Substituting the amine group with N-linked thioureas as in 11 and 12 improved 2-fold the KAs towards hCA I with respect to histamine. Among the remaining derivatives, only the bis-imidazole 16 reported an improved KA over the lead towards this isoform (KA of 0.72 µM). In fact, the presence of S-linked thioureas worsened the activation efficacy 2- (14, KA of 4.25 µM) or 4-fold (13, KA of 8.79 µM) with respect to the lead histamine. Oddly, the S-linked thiourea 19 showed a 2-fold improved KA compared to histamine, although bearing a dimethylamino group in place of the imidazole ring. As an exception, the N-rich compound 24 interestingly activated hCA I just two times less than histamine, in spite of a completely diverse structure. On the contrary, all histamine derivatives here reported showed a superior hCA II activation efficacy with respect to the lead (KA > 100 μM). Among mostly low micromolar CAAs (KAs in the range 2.1–13.5 μM), derivatives 3, 4 and 7 stood out as the most potent hCA II modulators of the study (KAs in the range 82 nM–0.57 μM). In particular, the α-methylation of histamine, as in 4, induced the largest increase of efficacy, up to a KA of 82 nM, when compared to the ineffective (as CAA) lead molecule.
- No submicromolar KA values was measured for 1–30 as hCA IV activators. Indeed, all KAs are in a rather flat low micromolar range (KAs in the range 1.02–13.9 μM), making this membrane-associated isozyme the less activated one by the compounds tested in this work. Interestingly, with the exception of compound 18, all derivatives were more efficient CAAs than the lead histamine, which showed a KA of 25.3 μM. Of note, the imidazole/thiazole swap led to the most effective activation increase with respect to the lead, with a KA of 1.02 μM in case of derivative 6.
- The other cytosolic isoform investigated here, hCA VII, was the most effectively activated one by the compounds investigated in this study. Indeed, a wide subset of KAs were detected in a submicromolar range (from 0.10 to 45.5 μM) for some of these derivatives. All of them showed much better activation profile than the reference compound histamine (KA of 37.5 μM) towards hCA VII. Contrariwise to hCA I and II, the most efficient CAAs were not detected among the methylhistamine derivatives 2–5: Nτ-methylhistidine 7, N-methylpiperidine 8 and the aryl ether 10, showed KAs ranging between 110 and 190 nM. The bis-imidazole 16 stood out as the most effective hCA VII activator of the study with a KA of 100 nM. Intriguingly, compound 23 did not activate hCA VII below 100 μM, whereas derivative 30, previously classified among the inactive compounds for the other CA isoforms, weakly activated this CNS-associated CA (KA of 5.06 μM). In fact, this isoform is one of the most widely spread in the brain, probably being involved in crucial metabolic/pH regulation processes, while it is not expressed in other tissues. It is thus relevant that a rather wide set of compounds was detected here (8, 9, 10, 13, 16), which showed a promising isoform selectivity towards hCA VII over the ubiquitous CAs (up to 100-fold over hCA II).

Table 2 also include the literature references regarding the compounds ability to cross the BBB, which presumably should also lead to brain CA activating effects, as well as evidences for their action at central level.

## 4. Conclusions and future perspectives

In the present study, we investigated the CA activating effects of a series of histamine receptors agonists/antagonists (compounds **2–30** in Figure 2) towards four hCA isoforms expressed in human brain, that are CA I, II, IV and VII. Though all derivatives possess moieties which can in principle shuttle protons in the pH range of 6–8, a consistent subset of them (**17**, **20**, **22**, **25–30**), not having the histamine chemotype in their structure, did not report any activation effect for the tested CA isoforms (K_A_s >100 µM). hCA I and II were effectively activated by methylhistamine derivatives (**2–7**), whereas more intriguing SAR were observed for hCA VII with more lipophilic groups (as in **6**, **8** or **10**) promoting greater and more selective isoform activation. Of note, a subset of selective hCA VII activators was identified, that could serve to drive the identification and optimisation of new brain specific CAAs. We are currently witnessing a second youth period for CAAs, because of innovative pharmacological studies spurring researchers to take into account these lately neglected agents for their potential clinical relevance in the treatment of emotional memory disorders, including the improvement of the clinical efficacy of exposure-based treatments of obsessive-compulsive disorders, phobias, generalised anxiety, and post-traumatic stress disorder. The here gathered data might also provide more insights on the pharmacodynamics of therapeutically used histamine modulators, whose therapeutic action and/or side effects could be related to polypharmacology. Overall, this work might bring new lights on the intricate relationship between CA activation and brain physiology.
